# Supplementary material for: Broadening participation in science through arts-facilitated experiences at a cultural festival
Source: PLoS One. 2023 May 8;18(5):e0284432. doi: 10.1371/journal.pone.0284432 (PMC10166484; doi:10.1371/journal.pone.0284432)
Supplement: Attachment — Submitted filename: Response to Reviewers.docx [file pone.0284432.s001.docx]

When submitting your revision, we need you to address these additional requirements.

1.Please ensure that your manuscript meets PLOS ONE's style requirements, including those for file naming. The PLOS ONE style templates can be found at

[https://journals.plos.org/plosone/s/file?id=wjVg/PLOSOne_formatting_sample_main_body.pdf](https://nam04.safelinks.protection.outlook.com/?url=https%3A%2F%2Fjournals.plos.org%2Fplosone%2Fs%2Ffile%3Fid%3DwjVg%2FPLOSOne_formatting_sample_main_body.pdf&data=05%7C01%7Ckari.oconnell%40oregonstate.edu%7C008af4348ceb4e0d773d08da90f3cbbc%7Cce6d05e13c5e4d6287a84c4a2713c113%7C0%7C0%7C637981674781830480%7CUnknown%7CTWFpbGZsb3d8eyJWIjoiMC4wLjAwMDAiLCJQIjoiV2luMzIiLCJBTiI6Ik1haWwiLCJXVCI6Mn0%3D%7C3000%7C%7C%7C&sdata=ZgSa8%2F9mbisZmed8Grng906S6tb2ej0zuaLUbEz9BgQ%3D&reserved=0) and

[https://journals.plos.org/plosone/s/file?id=ba62/PLOSOne_formatting_sample_title_authors_affiliations.pdf](https://nam04.safelinks.protection.outlook.com/?url=https%3A%2F%2Fjournals.plos.org%2Fplosone%2Fs%2Ffile%3Fid%3Dba62%2FPLOSOne_formatting_sample_title_authors_affiliations.pdf&data=05%7C01%7Ckari.oconnell%40oregonstate.edu%7C008af4348ceb4e0d773d08da90f3cbbc%7Cce6d05e13c5e4d6287a84c4a2713c113%7C0%7C0%7C637981674781830480%7CUnknown%7CTWFpbGZsb3d8eyJWIjoiMC4wLjAwMDAiLCJQIjoiV2luMzIiLCJBTiI6Ik1haWwiLCJXVCI6Mn0%3D%7C3000%7C%7C%7C&sdata=G1MqPDb2Mk044IIEMzXWVoD%2FUls5gUAR%2B1bxb1ogi0Q%3D&reserved=0).

Confirmed.

2. Please provide additional details regarding ethical approval and participant consent in the body of your manuscript. In the Methods section, please ensure that you have specified (1) the name of the IRB/ethics committee that approved your study, (2) whether consent was informed and (3) what type of consent you obtained (for instance, written or verbal). If your study included minors, state whether you obtained consent from parents or guardians. If the need for consent was waived by the ethics committee, please include this information.

The Oregon State University (OSU) Institutional Review Board (IRB) approved the study as an exempt study. The OSU IRB approved a waiver of documentation (signature) of informed consent. For paper surveys, we included brief study information and consent language at the top of the survey, and participants gave their consent by filling out part or all of the paper survey and turning it into the box (labeled as such). For interviews, we read study information to potential participants before starting the interview, allowing for verbal consent. Our study only included adults (18+), and as part of the verbal consent we included the requirement to be 18 to be part of the study.

Text to this effect now appears in section 2.3.

3. Thank you for stating the following in the Competing Interests section:

“I have read the journal's policy and the authors of this manuscript have the following competing interests: Mark Rosin is a part owner of Guerilla Science Global and sits on its Board of Directors. The remaining authors declare that there are no conflicts of interest”

Please confirm that this does not alter your adherence to all PLOS ONE policies on sharing data and materials, by including the following statement: ""This does not alter our adherence to PLOS ONE policies on sharing data and materials.” (as detailed online in our guide for authors [http://journals.plos.org/plosone/s/competing-interests](https://nam04.safelinks.protection.outlook.com/?url=http%3A%2F%2Fjournals.plos.org%2Fplosone%2Fs%2Fcompeting-interests&data=05%7C01%7Ckari.oconnell%40oregonstate.edu%7C008af4348ceb4e0d773d08da90f3cbbc%7Cce6d05e13c5e4d6287a84c4a2713c113%7C0%7C0%7C637981674781830480%7CUnknown%7CTWFpbGZsb3d8eyJWIjoiMC4wLjAwMDAiLCJQIjoiV2luMzIiLCJBTiI6Ik1haWwiLCJXVCI6Mn0%3D%7C3000%7C%7C%7C&sdata=7r2E4uAqR6utZJ1LfI1MN0dqrQkgJYFQL2LUmhGE2L0%3D&reserved=0)). If there are restrictions on sharing of data and/or materials, please state these. Please note that we cannot proceed with consideration of your article until this information has been declared.

Please include your updated Competing Interests statement in your cover letter; we will change the online submission form on your behalf.

Confirmed. Statement is now added. See updated cover letter.

4. In your Data Availability statement, you have not specified where the minimal data set underlying the results described in your manuscript can be found. PLOS defines a study's minimal data set as the underlying data used to reach the conclusions drawn in the manuscript and any additional data required to replicate the reported study findings in their entirety. All PLOS journals require that the minimal data set be made fully available. For more information about our data policy, please see [http://journals.plos.org/plosone/s/data-availability](https://nam04.safelinks.protection.outlook.com/?url=http%3A%2F%2Fjournals.plos.org%2Fplosone%2Fs%2Fdata-availability&data=05%7C01%7Ckari.oconnell%40oregonstate.edu%7C008af4348ceb4e0d773d08da90f3cbbc%7Cce6d05e13c5e4d6287a84c4a2713c113%7C0%7C0%7C637981674781830480%7CUnknown%7CTWFpbGZsb3d8eyJWIjoiMC4wLjAwMDAiLCJQIjoiV2luMzIiLCJBTiI6Ik1haWwiLCJXVCI6Mn0%3D%7C3000%7C%7C%7C&sdata=7F0xxiLfHZ4e9HQw2RKRMIyp2m%2BpPQWCL8atxCb0QZY%3D&reserved=0).

Upon re-submitting your revised manuscript, please upload your study’s minimal underlying data set as either Supporting Information files or to a stable, public repository and include the relevant URLs, DOIs, or accession numbers within your revised cover letter. For a list of acceptable repositories, please see [http://journals.plos.org/plosone/s/data-availability#loc-recommended-repositories](https://nam04.safelinks.protection.outlook.com/?url=http%3A%2F%2Fjournals.plos.org%2Fplosone%2Fs%2Fdata-availability%23loc-recommended-repositories&data=05%7C01%7Ckari.oconnell%40oregonstate.edu%7C008af4348ceb4e0d773d08da90f3cbbc%7Cce6d05e13c5e4d6287a84c4a2713c113%7C0%7C0%7C637981674781830480%7CUnknown%7CTWFpbGZsb3d8eyJWIjoiMC4wLjAwMDAiLCJQIjoiV2luMzIiLCJBTiI6Ik1haWwiLCJXVCI6Mn0%3D%7C3000%7C%7C%7C&sdata=Z4zT8R5p0vw77VmCHRP%2F2oKYUD1OSvbQWIuz9z7Df9c%3D&reserved=0). Any potentially identifying patient information must be fully anonymized.

Important: If there are ethical or legal restrictions to sharing your data publicly, please explain these restrictions in detail. Please see our guidelines for more information on what we consider unacceptable restrictions to publicly sharing data: [http://journals.plos.org/plosone/s/data-availability#loc-unacceptable-data-access-restrictions](https://nam04.safelinks.protection.outlook.com/?url=http%3A%2F%2Fjournals.plos.org%2Fplosone%2Fs%2Fdata-availability%23loc-unacceptable-data-access-restrictions&data=05%7C01%7Ckari.oconnell%40oregonstate.edu%7C008af4348ceb4e0d773d08da90f3cbbc%7Cce6d05e13c5e4d6287a84c4a2713c113%7C0%7C0%7C637981674781830480%7CUnknown%7CTWFpbGZsb3d8eyJWIjoiMC4wLjAwMDAiLCJQIjoiV2luMzIiLCJBTiI6Ik1haWwiLCJXVCI6Mn0%3D%7C3000%7C%7C%7C&sdata=pUn2u7V3iK1xJlNK%2FvtAB37ucSNRXsgaaw1PwGBm2TE%3D&reserved=0). Note that it is not acceptable for the authors to be the sole named individuals responsible for ensuring data access.

We will update your Data Availability statement to reflect the information you provide in your cover letter.

The data set on which this research is based is now publicly available at the following URL, hosted by the Scholars Archive at Oregon State University:

<https://doi.org/10.7267/hx11xq17s>

A statement to this effect is now included in the cover letter. The data is also now cited directly in the paper.

5. We note that you have indicated that data from this study are available upon request. PLOS only allows data to be available upon request if there are legal or ethical restrictions on sharing data publicly. For more information on unacceptable data access restrictions, please see [http://journals.plos.org/plosone/s/data-availability#loc-unacceptable-data-access-restrictions](https://nam04.safelinks.protection.outlook.com/?url=http%3A%2F%2Fjournals.plos.org%2Fplosone%2Fs%2Fdata-availability%23loc-unacceptable-data-access-restrictions&data=05%7C01%7Ckari.oconnell%40oregonstate.edu%7C008af4348ceb4e0d773d08da90f3cbbc%7Cce6d05e13c5e4d6287a84c4a2713c113%7C0%7C0%7C637981674781830480%7CUnknown%7CTWFpbGZsb3d8eyJWIjoiMC4wLjAwMDAiLCJQIjoiV2luMzIiLCJBTiI6Ik1haWwiLCJXVCI6Mn0%3D%7C3000%7C%7C%7C&sdata=pUn2u7V3iK1xJlNK%2FvtAB37ucSNRXsgaaw1PwGBm2TE%3D&reserved=0).

In your revised cover letter, please address the following prompts:

a) If there are ethical or legal restrictions on sharing a de-identified data set, please explain them in detail (e.g., data contain potentially sensitive information, data are owned by a third-party organization, etc.) and who has imposed them (e.g., an ethics committee). Please also provide contact information for a data access committee, ethics committee, or other institutional body to which data requests may be sent.

b) If there are no restrictions, please upload the minimal anonymized data set necessary to replicate your study findings as either Supporting Information files or to a stable, public repository and provide us with the relevant URLs, DOIs, or accession numbers. For a list of acceptable repositories, please see [http://journals.plos.org/plosone/s/data-availability#loc-recommended-repositories](https://nam04.safelinks.protection.outlook.com/?url=http%3A%2F%2Fjournals.plos.org%2Fplosone%2Fs%2Fdata-availability%23loc-recommended-repositories&data=05%7C01%7Ckari.oconnell%40oregonstate.edu%7C008af4348ceb4e0d773d08da90f3cbbc%7Cce6d05e13c5e4d6287a84c4a2713c113%7C0%7C0%7C637981674781830480%7CUnknown%7CTWFpbGZsb3d8eyJWIjoiMC4wLjAwMDAiLCJQIjoiV2luMzIiLCJBTiI6Ik1haWwiLCJXVCI6Mn0%3D%7C3000%7C%7C%7C&sdata=Z4zT8R5p0vw77VmCHRP%2F2oKYUD1OSvbQWIuz9z7Df9c%3D&reserved=0).

We will update your Data Availability statement on your behalf to reflect the information you provide.

The data set on which this research is based is now publicly available at the following URL, hosted by the Scholars Archive at Oregon State University:

<https://doi.org/10.7267/hx11xq17s>

A statement to this effect is now included in the cover letter. The data is also now cited directly in the paper.

6. Please include your full ethics statement in the ‘Methods’ section of your manuscript file. In your statement, please include the full name of the IRB or ethics committee who approved or waived your study, as well as whether or not you obtained informed written or verbal consent. If consent was waived for your study, please include this information in your statement as well.

The updated text has been added to the manuscript in the penultimate paragraph to section 2.3.

[Note: HTML markup is below. Please do not edit.]

Reviewers' comments:

Reviewer's Responses to Questions

**Comments to the Author**

1. Is the manuscript technically sound, and do the data support the conclusions?

The manuscript must describe a technically sound piece of scientific research with data that supports the conclusions. Experiments must have been conducted rigorously, with appropriate controls, replication, and sample sizes. The conclusions must be drawn appropriately based on the data presented.

Reviewer #1: Yes

2. Has the statistical analysis been performed appropriately and rigorously?

Reviewer #1: Yes

3. Have the authors made all data underlying the findings in their manuscript fully available?

The [PLOS Data policy](https://nam04.safelinks.protection.outlook.com/?url=http%3A%2F%2Fwww.plosone.org%2Fstatic%2Fpolicies.action%23sharing&data=05%7C01%7Ckari.oconnell%40oregonstate.edu%7C008af4348ceb4e0d773d08da90f3cbbc%7Cce6d05e13c5e4d6287a84c4a2713c113%7C0%7C0%7C637981674781986622%7CUnknown%7CTWFpbGZsb3d8eyJWIjoiMC4wLjAwMDAiLCJQIjoiV2luMzIiLCJBTiI6Ik1haWwiLCJXVCI6Mn0%3D%7C3000%7C%7C%7C&sdata=2A8VTDvuu8AscrG3onGEI0jIq8nOepvSaSz3Ry8h%2B%2Fs%3D&reserved=0) requires authors to make all data underlying the findings described in their manuscript fully available without restriction, with rare exception (please refer to the Data Availability Statement in the manuscript PDF file). The data should be provided as part of the manuscript or its supporting information, or deposited to a public repository. For example, in addition to summary statistics, the data points behind means, medians and variance measures should be available. If there are restrictions on publicly sharing data—e.g. participant privacy or use of data from a third party—those must be specified.

Reviewer #1: Yes

4. Is the manuscript presented in an intelligible fashion and written in standard English?

PLOS ONE does not copyedit accepted manuscripts, so the language in submitted articles must be clear, correct, and unambiguous. Any typographical or grammatical errors should be corrected at revision, so please note any specific errors here.

Reviewer #1: Yes

5. Review Comments to the Author

Please use the space provided to explain your answers to the questions above. You may also include additional comments for the author, including concerns about dual publication, research ethics, or publication ethics. (Please upload your review as an attachment if it exceeds 20,000 characters)

Reviewer #1: Well-written, clear, and engaging, this article contains an in situ example of people's interaction with an art-framed, science-related experience. The step by step logic of this work results in a substantial scope of data, with testing of assumptions and alternative interpretations built into the design. Given this, my comments are minimal and largely conceptual in nature.

Whether these "serendipitous" (line 508) interactions produce longer-term science persistence or identity is an open question, as the authors are aware. The clarity of this manuscript shows that a broader audience can be reached through this type of approach, which supports pursuing follow-up questions about behavior, perception, and identity development for those not interested nor strictly expecting to engage in science. Yet, people's relationship to science and their science identity is likely more nuanced than the broad way it was measured here. The term "science" may be relatively fixed in people's minds, whereas their actual interaction with science is varied and potentially deep. For example, experience in building, cooking, or gardening involves many scientific processes although may not be viewed as "doing science" by someone by a strictly topic-based definition of science.

All this said, my comment is about whether the authors are hoping these types of events will help broaden how people define science (science can contain an element of art, culture, etc.) or whether it is about deepening someone's interest in "science" as a more fixed area of study. There are assumptions that the authors may be making about the line between science and art as two disciplinary practices, or set of topics, that I'd like to hear more about.

*This is an excellent question, and we thank the reviewer for raising it.*

*We do indeed expect people’s relationship to science and their science identity to be more nuanced than the categorizations adopted in this article. Because the fieldwork took place in a loud, dynamic, busy environment in which survey participants had short attention spans, we chose, building on the associated research literature (see section 2.4, paragraph 1), to adopt the short non-intrusive questionnaires detailed in the Appendixes. It is an unfortunate feature of the kind of limited-survey, self-reporting empirical research conducted here that people’s interpretations of the questions, and the terms therein, such as “science” are inaccessible to us as researchers. (Furthermore, the same is true of the other interest and identity-based categories listed, for example, “politics”.)*

*Nevertheless, as the reviewer helpfully points out, it is important to make explicit in the article the associated underlying assumptions that follow from this fact. These assumptions, now stated in section 2.4, are that any variance in how the audience interprets terms like “science” will, on average, cancel out for a sufficiently large statistical sample. That is, on average, for every participant who interprets science in a narrow topic-based sense, another participant interprets it in a broader sense to include building, cooking, and gardening. In the absence of any systematic bias in the participants, this assumption is valid for the N>200 samples used here.*

*One potential source of systematic bias in the data is suggested by earlier research [1] that showed that people who are engaging in authentic scientific research (through a citizen science process) do not identify their engagement as being “with science” unless they are explicitly told so. Nevertheless, in the absence of more concrete data to indicate this may be the case here, we retain our assumption above, while making it explicit by discussing this in the article directly.*

*Text added in section 2.4:*

*In conducting our statistical analysis, we ignore the possibility of any systematic bias in how participants interpret the term science, be it in a narrow topic-based sense or an expansive sense to include activities such as cooking or gardening. Earlier work has suggested that labeling an activity as "science" may have to be explicit for participants to change how they self-report their engagement with science [1].*

*[1] Brossard, Dominique, Lewenstein, Bruce V., & Bonney, Rick. (2005, 15 July). Scientific Knowledge and Attitude Change: The Impact of a Citizen Science Project. International Journal of Science Education, 27(9), 1099-1121.*
