# Supplementary material for: Broadening participation in science through arts-facilitated experiences at a cultural festival
Source: PLoS One. 2023 May 8;18(5):e0284432. doi: 10.1371/journal.pone.0284432 (PMC10166484; doi:10.1371/journal.pone.0284432)
Supplement: Attachment — Submitted filename: Response to Reviewers 2.docx [file pone.0284432.s002.docx]

Dear Editor, Reviewers,

We thank the reviewers for their attentive and insightful comments.

We found reviewer #2’s comments to be extremely helpful in highlighting some areas where a lack of clarity upon the authors’ part was leading to confusion. Indeed, in pointing these out the authors were able to hone their argument and create a clearer, more readable text. Details of how we have modified our manuscript are provided below as in-line responses to the reviewer’s comments.

We found reviewer #3’s comments to be supportive and encouraging. Their interpretation of our work was in total alignment with the message we were trying to convey. There were no specific recommendations, and so we simply thank the reviewer for their time in providing such a close reading.

We hope our responses succeed in addressing the reviewers’ concerns, where they existed.

Sincerely,

Mark Rosin, on behalf of the author team.

**Reviewer 2:**

**Reviewer #2:** This is an interesting article that aims to broaden participation to science using a festival or carnival mode. The authors argue this is an emancipatory approach to learning, and also improves access and inclusion. While informal learning opportunities are valuable, many that are clearly science-identified may be missed by people who do not see themselves as science engaged, and therefore may miss out.

**Response**: We thank the reviewer for this thorough and insightful review. There were several excellent points that we have adapted the paper to address, and additional stylistic recommendations that are appreciated.

**Reviewer #2:** The main challenge I see in the piece right now is that the framework and “theory of change” is somehow scattered and embedded throughout the manuscript.

The authors would improve the manuscript by bringing the framework forward (or at least foreshadowing it) earlier on page 4. Specifically, the explanation on page 7 is compelling.

**Response**: This is an excellent suggestion. We have made explicit our Theory of Change, now at the top of page 5. We also foreground an associated reference for the reader that would like more details on the Theory of Change.

Activities: Live creative public science experiences in cultural and community spaces.

Outputs: People who would not otherwise engage with science build a connection with it.

Outcomes: People’s lives are enriched through meaningful and relevant relationships with science.

**Reviewer #2:** However, I am trying to reconcile this with being “stealth” which is a word used in the research question. Stealth assumes trying to “hide” the intent of science, kind of like hiding veggies in a dish and surprising the person later that they ate veggies. Pop up implies unplanned, which can help people impromptu walk into something they might not otherwise intend to engage in. Neither of these is as clearly “emancipatory” in the way a festival may promote community engagement and a broader invitation. This is revisited at bottom of page 28 and page 34 (carnival mode as emancipatory).

**Response**: This is an excellent point! Thanks to the reviewer.

We feel there are two separate, but related, points: First, the emancipatory nature of carnival is indeed different from that of Guerilla Science events. Second, it is not obvious how emancipation is related to an event being pop-up. Let us address these

First, we are indeed, implicitly, using two different meanings of emancipation: Festivals and carnival, a la Bhaktin, are emancipatory spaces where participants can let off steam, explore, and learn through play, as described on p7 (for more details see Appendix 2 of Bevan B, Mejias S, Rosin M, Wong J. The main course was mealworms: The epistemics of art and science in public engagement. Leonardo. 2021 Aug 9;54(4):456-61). On the other hand, the impact of the Guerilla Science project is emancipatory in a more narrowly defined science-literacy-building sense. Guerilla Science’s emancipatory impact is more closely rooted in the type of broad, and sometimes radical, educational benefits espoused by Dewey and Friere: democratizing and critical education. This is discussed in earlier work *Rosin M, Wong J, O’Connell K, Storksdieck M, Keys B. Guerilla Science: Mixing science with art, music and play in unusual settings. Leonardo. 2021 Apr 15;54(2):191-5*.

This distinction is now made explicit on page 9.

Second, the role of pop-up or stealth: The pop-up or stealth nature of the event is important in engaging people who would not otherwise self-select into an event where science is present. It is a mechanism through which the Guerilla Science activities can be situated in physical proximity to new audiences. It is a necessary, but not sufficient condition for broadening participation. This is what we are talking about when we refer to *access.*

Now, when this physical proximity is combined with event design or format or created context that resonates with the individuals’ learning identities, then this can lead to a broadening participation effect. This is what we are talking about when we refer to *inclusion* and is discussed at the towards of the end of section 2.1:

“Thus we anticipate that festivals may address the issue of inclusiveness, by representing opportunities for people of multiple identities to connect with a wide range of activities.”

The role of festivals, specifically their emancipatory power, is largely related to the inclusive aspect of these two components. At a festival, people feel freer to participate. They try on new roles. They can feel themselves more open to being included. In the context of Guerilla Science, this means we are offering ways to engage with science that are different from the typical, that do not require embracing or liking science, by offering engagement mechanisms that align with non-science (learner, enthusiast) identities. In that sense, through the context (festival) and the delivery mechanisms (art- and design-based inventive and sometimes outrageous means to explore scientific phenomena) we free a person from engaging with science in formats that are typical for science engagement. That is, one emancipatory element is the form and context of the engagement format.

This is re-emphasized on page 8.

**Reviewer #2:** As a reader who is pretty familiar with the power of informal learning experiences, the authors need to put a sharper point on what’s new from this study…and to make it seem less like an evaluation of this activity.

**Response**: What we consider to be new: the question on whether arts/design-infused different forms of science engagement actually attract new audiences or simply lead to a cherry-picked self-selection. This question has not been answered well or with strong empirical evidence, and as far as we know, this is only the second such study, one that confirms our prior findings from a different context (a music festival that took place at a remote location and required considerable financial resources and time to attend). (b) One could consider this research as an evaluation of Guerilla Science, or even more narrowly, as an evaluation of the specific way in which GS was implemented at Figment, or you can see it as applied research that advances the basic notion of whether approaches of this kind are promising practices for reaching new audiences. A strict evaluation might ask: Does the event resonate with audiences, is it of high quality, does it achieve its stated outcomes? An evaluation can also ask applied research questions: does the skillful implementation prove the underlying implied theoretical framework? We did the latter, and in doing so we studied the underlying phenomenon with a concrete example, one that prior evaluations had shown to be of high quality. We find the distinction between applied research and evaluation that tests underlying assumptions unnecessary. What matters is whether the research questions are of interest, matter to people, and are based on solid theoretical grounding. We believe we did that. We also want to point out that many research studies in education study a phenomenon using a concrete implementation of some theory-driven intervention. We did exactly that; in fact, the proposal to the National Science Foundation spelled out our research approach and was funded as a “research-in-service-to-practice” study.

**Reviewer #2:** Toward this end, the discussion could put forward ideas of the kinds of exhibits or festivals that could help citizen science grab hold of our public.

**Response**: We also conduct research on citizen science, and are not entirely sure whether or how the concept of Guerilla Science connects with citizen science. We acknowledge that not all citizen science markets itself as such and other terms are sometimes used, such as public participation in scientific research, participatory science, or community science, and we acknowledge that not all projects who claim to be “citizen science” actually seek to make contributions to research. This benefit of citizen science in terms of engaging the public in research rests on the notion that people realize they are doing science; in fact, research shows that people need to be reminded of it when they engage in citizen science or else they may not reap the full benefits. For example, it was shown in a study by Brossard et al, that in the case they studied people didn't learn science, in part because they didn't know they were doing science.[Brossard, D., Lewenstein, B. V., & Bonney, R. (2005). Scientific Knowledge and Attitude Change: The Impact of a Citizen Science Project. International Journal of Science Education, 27(9), 1099-1121.] The issue is therefore almost inverted from Guerilla Science: Citizen Science can be seen as providing a science-interested and supportive public with opportunity to partake in science in a contributory fashion while Guerilla Science provides those who may not otherwise choose to engage with science with ways to engage that are less “sciency”.

Prompted by this reviewer question, we have now introduced a brief discussion at the bottom of page 17 related to narrow and expansive descriptions of science, and cited the paper mentioned above.

**Reviewer #2:** The second suggestion is some kind of recognition that a STEAM movement already exists- so perhaps some brief contextualization about how guerilla science differs from STEAM. This is on page 4.

**Response**: This is a good point. We have added a short section just before the paper’s conclusion explaining our stance on “STEAM” as a descriptor of this kind of work, and added a review-style paper reference that supports our stance.

**Reviewer #2:** There is also interesting data presented by the authors and by the personal communication of Jon Miller in Table 9 that suggest that science museum and art gallery attendees might be similar people. I think more could be made of this prospect, possibly in the introduction and then in the discussion.

**Response**: The Table 9’s main message is twofold: High participation at the national level in cultural events, whether science- or art-related, is low, that is, only a small segment of a representative sample of US residents frequently engage in either. However, our sample of mostly New York-based residents (those who visited Figment) shows a much higher average rate of art- and science-participation than the US public. Assuming NY residents don’t have a much higher interest in science, their higher participation in spare-time science activities might be a reflection of the overall higher participation in cultural activities by New Yorkers. We do not claim, nor does the table suggest as the reviewer stated, that the people who participate in art-related activities may also participate in science-related activities. Miller was not able to cross-tabulate the data for us. In short, we conclude from Table 9 that cultural participation in NY is higher than in the US on average (not surprising, but worth showing). And yes, there are certainly individuals who are broadly culturally engaged and may frequent both, an art and a natural history museum. However, Guerilla Science in general (and our research in particular) was not designed to target the group that may appreciate both. In contrast to the reviewer, we would see it as a distraction from our narrative, particularly in light of the fact that we do not have robust data on the size of the overlap.

**Reviewer #2**: Finally, small word choices could improve the readability of this article, particularly as the authors consider the opening of the article. There are some small word choices from the abstract through the first few pages that obscure the strength of this piece.

Abstract: Consider changing the word “choir” – in pages 3-4 you do a great job of explaining self-selection. Consider a word choice that brings forward the idea of self-selection. Those unfamiliar with choir metaphor may be confused by this in the abstract.

**Response**: We cut the term “choir” from the abstract.

**Reviewer #2:** Page 3, Line: 36-37—That is, audiences who do not actively seek out science learning opportunities. This is a fragment- and should be connected to the previous sentence.

**Response**: A well made point. We added a semicolon

**Reviewer #2:** This challenge is motivated by two needs. Possibly rephrase: Addressing this challenge relies upon two principles.

**Response**: Good suggestion. We have modified.

**Reviewer #3**

**Reviewer #3:** This study looks at the complicated setting of informal science learning to better understand design strategies for reaching broader audiences, defined by the authors as audiences with less access or inclusion in participating in science engagement.

**Response**: We thank the reviewer for this helpful summary, and are pleased that the salient points of our argument resonated.

**Reviewer #3:** The study design compares the general visitor to a regional arts festival to those who participate in a specific area of the festival- that incorporate stealth science experiences. It uses descriptive statistics to understand if the intervention reaches the desired broader audiences or if the experience results in self-selection by those with pre-existing science affinity. And builds on prior festival work, this time eliminating two access barriers (cost is free and public transportation is plentiful) while maintaining the stealth experiences that integrate arts and science expertise and content.

The authors cite broader impacts and inclusion as typically addressing race, income, ability, and gender but, in this study, broader audiences are visitors outside the average museum-going behavior. Data show that this arts festival in fact draws a higher than average group of cultural event visitors (science and art) but with more arts aligned than science aligned, yet all had positive outcomes from the stealth experience.

These experiences and this context were shown to be socially motivated, people came in groups, for a fun, active experience with an expectation to learn something, and in the stealth experience, these expectations were met and resulted in higher interest in science as part of that learning.

This study sheds light on our expectations about audiences in different contexts. The authors cite limitations to prior studies in terms of access, this study illuminates other assumptions about who attends a festival (compared to a museum or designated informal science learning context. Was this arts festival a draw to “culture vultures” more than other festivals might be?

Similar studies in an expanding variety of contexts for instance, county fairs; music or dance festivals centered in specific cultural communities; or local sports events where people gather to celebrate the effort of community members, each could have similar stealth experiences built in that teach us how people connect their local activities to science, art and social goals and questions.

This is a well-designed and written study that informs both festival and informal learning institutions in thinking about expectations and motivations as they attempt to engage visitors in science through different frames.
